# BCI move: exploring pediatric BCI-controlled power mobility

**DOI:** 10.3389/fnhum.2025.1456692

**Published:** 2025-04-09

**Authors:** Leah Hammond, Danette Rowley, Corinne Tuck, Erica Danielle Floreani, Amy Wieler, Vella Shin-Hyung Kim, Hosein Bahari, John Andersen, Adam Kirton, Eli Kinney-Lang

**Affiliations:** ^1^Brain-Computer Interface Program, Imagination Centre, Glenrose Rehabilitation Hospital, Edmonton, AB, Canada; ^2^BCI4Kids, Cumming School of Medicine, University of Calgary, Calgary, AB, Canada; ^3^Department of Pediatrics, University of Alberta, Edmonton, AB, Canada; ^4^Department of Pediatrics, University of Calgary, Calgary, AB, Canada

**Keywords:** brain-computer interface, pediatrics, power mobility, cerebral palsy, alternative access technology, brain-machine interface

## Abstract

**Introduction:**

Children and young people (CYP) with severe physical disabilities often experience barriers to independent mobility, placing them at risk for developmental impairments and restricting their independence and participation. Pilot work suggests that brain-computer interface (BCIs) could enable powered mobility control for children with motor disabilities. We explored how severely disabled CYP could use BCI to achieve individualized, functional power mobility goals and acquire power mobility skills. We also explored the practicality of pediatric BCI-enabled power mobility.

**Methods:**

Nine CYP aged 7-17 years with severe physical disabilities and their caregivers participated in up to 12 BCI-enabled power mobility training sessions focused on a personalized power mobility goal. Goal achievement was assessed using the Canadian Occupational Performance Measure (COPM) and Goal Attainment Scaling (GAS). The Assessment for Learning Powered Mobility (ALP) was used to measure session-by-session power mobility skill acquisition. BCI set-up and calibration metrics, perceived workload, and participant engagement were also reported.

**Results:**

Significant improvements in COPM performance (*Z* = −2.869, adjusted *p* = 0.012) and satisfaction scores (*Z* = −2.809, adjusted *p* = 0.015) and GAS T scores (*Z* = −2.805, *p* = 0.005) were observed following the intervention. ALP scores displayed a small but significant increase over time (*R*^2^ = 0.07–0.19; adjusted *p* = <0.001–0.039), with 7/9 participants achieving increased overall ALP scores following the intervention. Setup and calibration times were practical although calibration consistency was highly variable. Participants reported moderate workload with no significant change over time (*R*^2^ = 0.00–0.13; adjusted *p* = 0.006–1.000), although there was a trend towards increased frustration over time(*R*^2^ = 0.13; adjusted *p* = 0.006).

**Discussion:**

Participants were highly engaged throughout the intervention. BCI-enabled power mobility appears to help CYP with severe physical disabilities achieve personalized power mobility goals and acquire power mobility skills. BCI-enabled power mobility training also appears to be practical, but BCI performance optimization and skill acquisition may be needed to translate this technology into clinical use.

## 1 Introduction

Independent mobility provides children with new opportunities to explore their environments, facilitating cognitive, perceptual, social, and communicative development (Adolph and Hoch, 2019; Campos et al., 2000; Iverson, 2010; Von Hofsten, 2009). Powered mobility devices (PMDs), such as wheelchairs or trainers powered by motors, provide opportunities for children and young people (CYP) with motor disabilities to experience independent mobility (Livingstone and Paleg, 2014; Livingstone and Field, 2015). Access to power mobility is considered medically necessary for such CYP, due to the wide range of developmental benefits it can enable (Guerette et al., 2013; Rosen et al., 2018). CYP with severe mobility limitations may require custom access methods for PMD operation, such as mechanical or proximity switches (Huang et al., 2014; Kenyon et al., 2015; Kenyon et al., 2017). However, it can be extremely demanding, fatiguing, or outright impossible to perform the physical actions required for reliable, accurate, and repetitive PMD control. These barriers can ultimately lead to CYP being unable to achieve the strict “functional driving skills” criteria required for funding eligibility for powerchair provisions (Kenyon et al., 2015). Without the ability to independently navigate their environment, these CYP face significant barriers to participation and are at risk for secondary developmental impairments, affecting cognition, perception, communication, language, social participation, play, and relationship development (Anderson et al., 2013; Livingstone and Field, 2014; Rosen et al., 2018), as well as reduced engagement, motivation, and self-confidence (Livingstone and Field, 2015) compared to their peers.

Brain-computer interfaces (BCIs) are an emerging alternative access solution for CYP with complex physical disabilities and needs (Jadavji et al., 2022; Kinney-Lang et al., 2023; Kelly et al., 2020; Kelly et al., 2023). BCIs bypass the body completely, and instead interpret the user’s intent by identifying changing patterns in their brain activity to exert control over an external device or application (Wolpaw et al., 2002). Most non-invasive BCIs use electroencephalography (EEG) to record changes in the brain’s electrical activity through sensors placed on the scalp. The acquired EEG signals are then processed to remove unwanted information and extract the relevant specific brain-patterns of interest through machine learning algorithms to identify the user’s intended output command (Wolpaw et al., 2020). There are several distinct control paradigms typically used to control BCI systems, such as attending to an external stimulus to elicit a specific brain response (e.g., a time-locked event-related potential like the P300-oddball response), or through self-paced manipulation of a neural response (e.g., through altering sensorimotor rhythms through motor imagery; Abiri et al., 2019). Despite the huge potential of BCI endorsed by both clinicians and families, CYP with severe disabilities have been almost entirely neglected from BCI research (Kinney-Lang et al., 2023; Kirton, 2023).

Typically developing children (Zhang et al., 2019) and children with cerebral palsy (Jadavji et al., 2021) are capable of controlling simple BCI systems. Children with quadriplegic cerebral palsy have also demonstrated the ability to use simple, noninvasive BCIs to accomplish varied personal goals in various environments, including at-home (Kelly et al., 2020; Floreani et al., 2022a; Kelly et al., 2023). Across an international pediatric collaborative network BCI-CAN (Kinney-Lang et al., 2020), families continue to identify independent mobility as a top priority for BCI-based applications, a sentiment mirrored by clinicians caring for persons with severe disabilities (Letourneau et al., 2020).

Control of PMDs through a BCI has been demonstrated for both neurotypical adults and adult end-users living with disabilities (Fernández-Rodríguez et al., 2016; Al-qaysi et al., 2018; Cruz et al., 2021; Tang et al., 2018). The method of control for these studies has ranged from direct mapping of BCI activations to directional control of the PMD (allowing the user to drive forward, turn or stop; Fernández-Rodríguez et al., 2016; Al-qaysi et al., 2018), to using the BCI for selecting a preset destination to which the PMD can navigate to without further input from the driver (Fernández-Rodríguez et al., 2016; Tang et al., 2018). Initial proof-of-concept studies have also demonstrated that children with quadriplegic cerebral palsy can use a simple BCI system to activate and move themselves forward in a PMD (Floreani et al., 2021), and report finding the BCI-enabled movement enjoyable (see Supplementary video). A pilot study demonstrated that PMD control through BCI as an alternative access method was feasible and well-tolerated by children with quadriplegic cerebral palsy, further emphasizing the potential for a longitudinal study on using BCIs for learning PMD control (Floreani et al., 2022b). Based on these early successes, family and CYP priorities, and the potential for impact, this work explored how severely disabled CYP can use BCI to achieve individualized, functional power mobility goals and realize new levels of independence and function.

## 2 Materials and methods

### 2.1 Participant criteria and recruitment

CYP with severe physical disabilities were recruited through clinical pediatric BCI research programs at two tertiary hospitals in Alberta, Canada (BCI4Kids at Alberta Children’s Hospital in Calgary (Jadavji et al., 2022); Imagination Centre BCI Program at the Glenrose Rehabilitation Hospital in Edmonton). Inclusion criteria were: (1) age 5–21 years; (2) non-ambulatory (Gross Motor Function Classification Score [GMFCS; Palisano et al., 1997] IV–V or equivalent) with minimal functional hand use (Manual Ability Classification Score [MACS; Eliasson et al., 2006] IV–V or equivalent); (3) estimated school age cognitive capacity based on evidence from parents and clinicians; (4a) no current method for accessing power mobility or (4b) current power mobility access method unable to achieve participant goals; and (5) informed consent/assent. To display evidence of appropriate cognitive capacity, CYP needed to be able to (1) remain awake and alert throughout a 1-h training session, (2) attend to a single activity for at least 10 min, (3) reliably answer yes/no questions using any communication method, and (4) follow simple verbal directions. CYP with severe epileptic encephalopathy, severe uncorrected visual or hearing impairment, or who were unable to attend in-person training sessions were excluded. Assent and parental consent were obtained in accordance with the Conjoint Health Research Ethics Board at the University of Calgary.

### 2.2 Equipment

#### 2.2.1 BCI systems

Participants used either an Emotiv Epoc X or an Emotiv Flex (*Emotiv, USA*) headset, depending on their individual needs. Both headsets non-invasively record electroencephalography (EEG) data from the scalp. The Epoc X is a 14-channel (AF3, F7, F3, FC5, T7, P7, O1, O2, P8, T8, FC6, F4, F8, AF4), saline-based, headband-style headset with a sampling rate of 128 Hz. The Emotiv Flex is a 32-channel, saline or gel-based cap-style headset, which allows for flexible 10–20 system sensor placement, and a sampling rate of 256 Hz. These BCI systems were selected based on their suitability for pediatric users (Jadavji et al., 2022). The cap-style Emotiv Flex was used for participants who experienced involuntary head movements or tremors, as it could be more securely attached than the headband-style Epoc X, preserving electrode placement and limiting the impact of movement artifacts. The gel-based Emotiv Flex system was used for a participant with thick, coarse hair to optimize sensor connectivity and preserve electrode placement. Where necessary, Emotiv Flex caps were modified with custom chin straps or stretchy caps placed over the headset to prevent shifting of the cap throughout the session.

#### 2.2.2 BCI-to-switch interface

BCI output commands were translated to control the PMD using Think2Switch (*Possibility Neurotechnologies, Canada*), a proprietary interface device that enables the wireless transmission of BCI commands from a computer to up to four standard 3.5 mm mono jack switch outputs. Each mono jack output can be mapped to drive commands (e.g., front, left, right, and reverse). Participants used BCI to control up to two drive commands based on their personalized goals (i.e., forward and left turn of a PMD). The Think2Switch software included tools to monitor EEG signal quality, run BCI calibration, adjust control parameters, toggle the BCI control on and off, and display visual feedback to the user. Where possible, a Microsoft Surface Pro tablet computer was mounted on the PMD to run the software and enable ongoing monitoring of visual feedback. In situations where the Surface Pro could not be mounted on the PMD, the Surface Pro was positioned on the participant’s wheelchair tray or carried beside the PMD by a member of research staff to ensure continuous connection for monitoring.

#### 2.2.3 Power mobility devices

**Figure 1:**
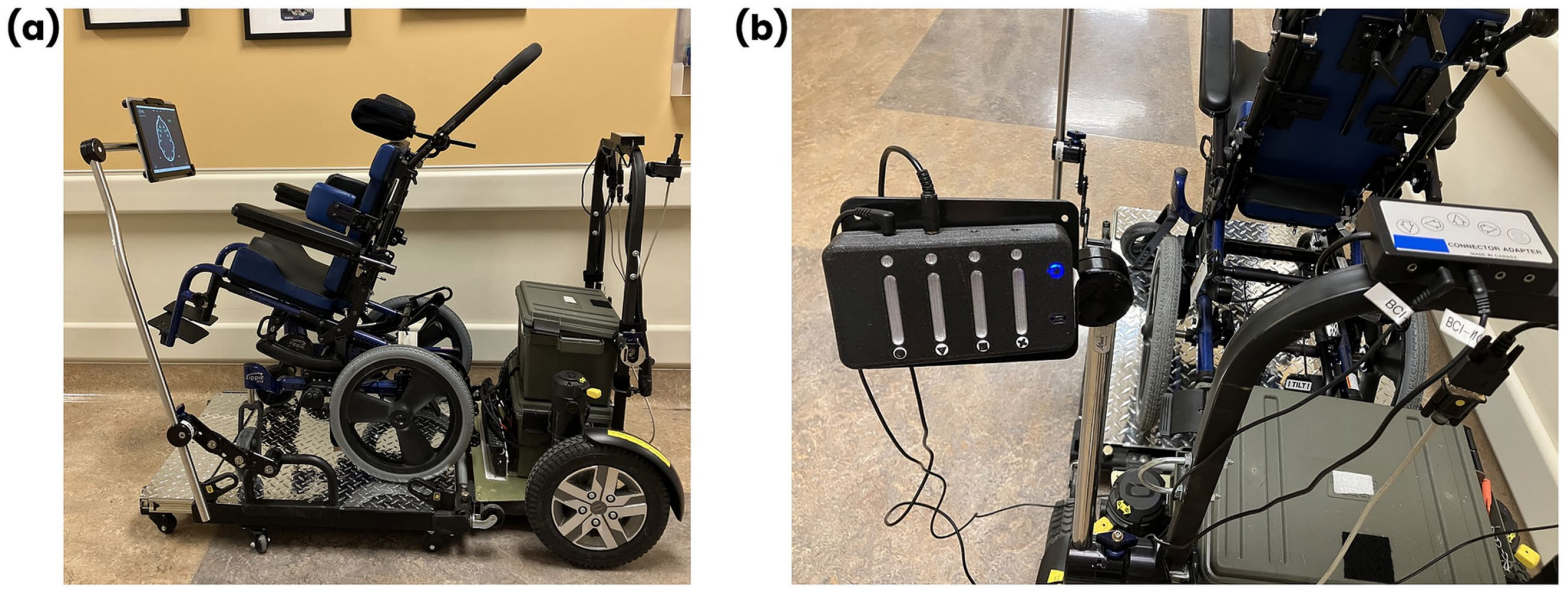
PMD setup. **(a)** The power mobility trainer device consists of a power wheelchair base, which propels a wheeled platform. A manual wheelchair can be rolled onto the platform, allowing participants to sit in their manual wheelchair and practice using a new access method. A Microsoft Surface Pro tablet was mounted on power mobility trainer, within the participants’ line of sight, to enable provision of visual feedback from the Think2Switch software. **(b)** The Think2Switch was mounted on the back of the power mobility trainer. The Think2Switch connects wirelessly to the BCI headset. Up to two mono jack outputs from the Think2Switch were connected to mono jack inputs on the power mobility trainer to control up to two drive commands.

Participants used either a switch-enabled power wheelchair or a power mobility trainer device. The trainer device consisted of a wheeled platform powered by a power wheelchair base enabling participants to be secured in the trainer within their own manual chair. The trainer was selected for participants who used custom seating in their own manual chair that could not easily be transferred to a power wheelchair and in cases where an appropriate fitting power wheelchair was unavailable. The PMDs used in this study could be controlled with standard switch inputs (3.5 mm mono jack inputs) to drive forward, left, right, and reverse. One to two Think2Switch mono jack outputs were connected to PMD inputs, allowing participants to control up to two drive commands using BCI. All participants began the intervention controlling one drive command using BCI. Participants’ neutral BCI command was used to maintain a stationary position or stop the PMD. An emergency stop switch was monitored for participant safety. Figure 1 demonstrates the set-up of the PMD and Think2Switch.

### 2.3 Study overview

**Figure 2:**
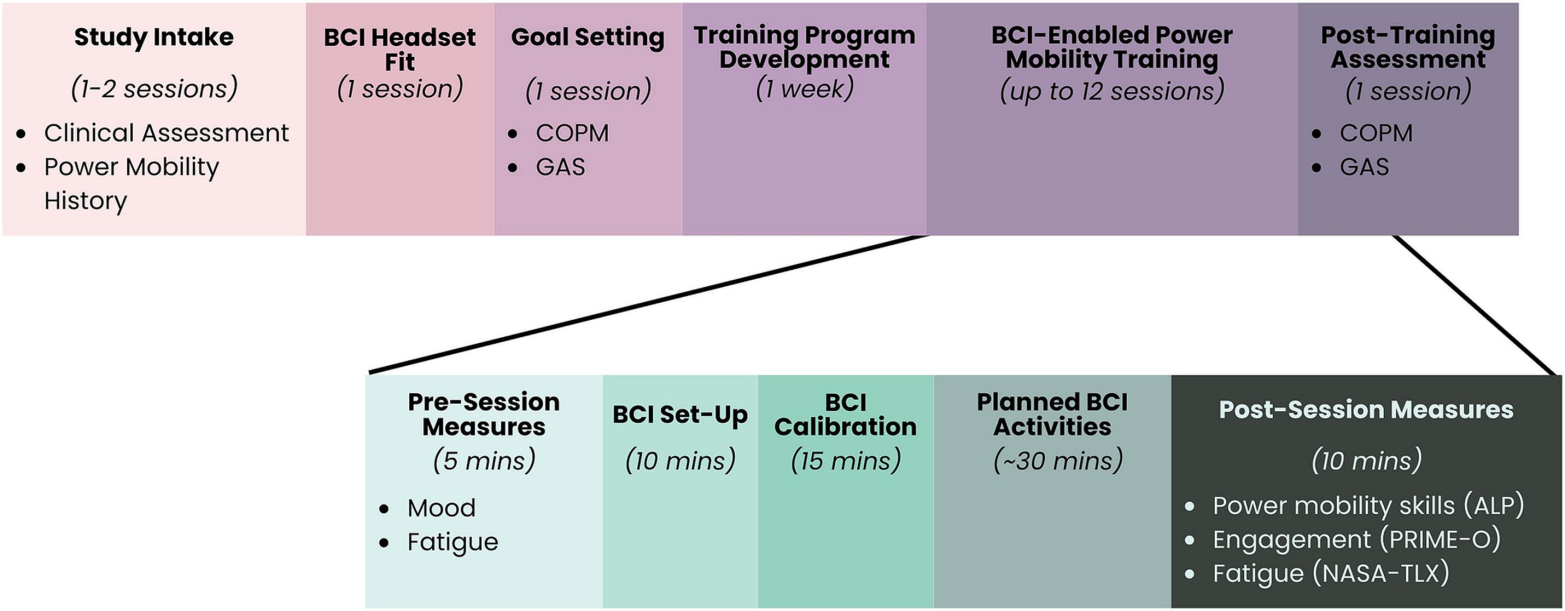
Study methods. ALP, Assessment for Learning Power Mobility (Nilsson and Durkin, 2014); COPM, Canadian Occupational Performance Measure (Law et al., 1990); GAS, Goal Attainment Scaling (Turner-Stokes, 2009); NASA-TLX, NASA Task Load Index (Hart and Staveland, 1988; Laurie-Rose et al., 2017); PRIME-O, Pediatric Rehabilitation Intervention Measure of Engagement – Observer Version (King et al., 2015).

Participants attended up to 12 BCI power mobility sessions at the Alberta Children’s Hospital (*Calgary, Canada*) or Glenrose Rehabilitation Hospital (*Edmonton, Canada*). A simple, commercial-grade BCI system (Emotiv EPOC X or Emotiv Flex; Emotiv, USA) and commercial-grade BCI-to-switch interface (Think2Switch; *Possibility Neurotechnologies*) was used to activate a power mobility device (power wheelchair or power mobility trainer). Participants and their families identified personalized power mobility goals to focus on over the course of training with the assistance of experienced therapists. During training sessions, participants completed play-based activities designed to promote power mobility skill development aligned with their personalized goal. Figure 2 provides a visual representation of the study design.

### 2.4 Screening and study intake

An occupational therapist collected a structured clinical history from each participant’s family, including previous use of power mobility and assistive technologies. The participant’s seating and positioning was also assessed to ensure optimal fit with the PMD and BCI headset. Screening was conducted during up to two 1-h sessions.

### 2.5 BCI headset fit

Based on clinical history, examination, and family discussions, potential BCI and power mobility systems were identified and trialed to ensure participant comfort and usability. Occupational therapists and families determined whether BCI would be used as the sole access method or if BCI would be combined with an existing access method for power mobility. Participants collaborated with research team clinicians to identify personalized power mobility commands and confirm candidate headset fit, tolerability, and ability to achieve adequate signal quality.

#### 2.5.1 BCI calibration

This study used a BCI paradigm based on imagined mental commands: participants repeatedly visualized a personally meaningful physical action or movement, like kicking a ball or pushing a sibling, to activate the BCI system. Mental commands and neutral states were trained in combination with visual feedback and/or auditory feedback. EEG data collected while executing the mental command were compared to EEG features collected at rest to create a classifier for real-time decoding of EEG features. BCI calibration was performed using Emotiv’s proprietary calibration software and classifiers were generated using Emotiv’s proprietary Cortex API. As such, the signal processing in this study including filtering, artifact rejection, and classification, rely upon the default output provided by the Cortex API as our signal processing pipeline. This approach using the Cortex API has been used a number of times previously when exploring low-cost EEG systems for simple control, including for wheel-chair control (Thwe et al., 2024). A single calibration trial consisted of 8 s of focused attention, either performing the selected mental command or resting for the neutral state. The mental command and the neutral state were each calibrated 6–12 times, based on participant tolerability and frustration and distinguishability of trained mental commands (based on visual feedback provided by Emotiv’s calibration software).

The Cortex API software generated a “consistency score” on a scale from 0 to 100% for each calibration trial, effectively comparing the current calibration trial to the preceding trial. This “consistency” measure is based on proprietary measurements within the Cortex API software, therefore it is unclear what metrics or features were used to determine trial-to-trial consistency. However, calibration guidelines provided by the developer (Emotiv) recommend that users try to maximize this consistency metric (i.e., as close to 100% as possible). As such, trials that scored poorly (<30%) were rejected and re-attempted unless the participant indicated that they had been generating their mental command. Trials with excessive participant movement or distraction were also rejected. After two attempts, re-attempted trials were accepted to continue session momentum and minimize participant frustration.

### 2.6 Goal setting and program planning

Participants’ self-reported COPM performance and satisfaction were the primary subjective outcomes for this study, with a change of 2 points or greater between baseline and post-intervention scores indicating clinical significance (Cusick et al., 2007). Occupational therapists completed the Canadian Occupational Performance Measure (COPM; Law et al., 1990) with participants and their caregivers to identify personal power mobility goals. Participants and their caregivers rated their baseline perceived performance, importance, and satisfaction on a scale from 1 to 10. If appropriate, additional goals were added throughout the intervention, as participants’ power mobility skills progressed over time.

Therapists applied Goal Attainment Scaling (GAS) to define observable and quantifiable subgoals for each personalized power mobility goal (Turner-Stokes, 2009). Baseline performance was set at −2 for all subgoals where participants had no previous experience with BCI and −1 for subgoals where participants had some previous experience with BCI. Therapists defined a range of outcomes for each subgoal, creating a 5-point scale that was used to evaluate individual changes following the intervention. Scales ranged from −2 to +2, where −2 is no change in performance from baseline, 0 is the expected goal, and +2 is much more than the expected goal (Turner-Stokes, 2009). Subgoal achievement ratings were then summarized into the GAS T score, which serves as an overall goal attainment score for each participant. A T score of 50 or higher corresponds to achievement of the expected goal and indicates a clinically significant changes in goal attainment (Turner-Stokes, 2009). Supplementary material 1 shows an example of GAS scoring for participants’ personalized power mobility goals.

Based on each participant’s personal power mobility goals, therapists developed an individualized power mobility training program. Training programs were comprised of up to 12, 60–90 min training sessions. Families were free to choose the number of sessions they completed, up to a maximum of 12, within the data collection period. Programs focused on progressive improvements to power mobility control through play-based and goal-directed activities tailored to each participant’s interests.

### 2.7 BCI-enabled power mobility training

At the start of each session, participants were asked to report their fatigue (*“How tired are you feeling today?”; 1* = *Very tired and 5* = *Wide awake*) and mood (*“How are you feeling today?”*). Visual supports were provided to facilitate participant responding. Participants were then positioned in the PMD, the EEG headset was applied, and the BCI system was calibrated, using the calibration approach described above. Then, participants carried out specific goal-related activities according to their individualized training plan. BCI calibration trials and EEG data were recorded for each session as well as goal-related data from session activities (e.g., distance traveled using a single mental command; length of time maintaining mental command below threshold). Where possible, sessions were video recorded to confirm goal-related data.

After each session, therapists evaluated each participant’s phase of power mobility learning using the Assessment of Learning Powered Mobility Use (ALP; Nilsson and Durkin, 2014). Participants are assigned a phase of learning on a scale from 1 (Novice) to 8 (Expert) across 5 observational categories: *Attention* (signs of attention regulation)*, Activity & Movement* (motor control and motor performance)*, Understanding of Tool Use* (observable cognitive components of power mobility use)*, Expressions & Emotions* (signs of motivation), and *Interaction & Communication* (social interplay). To guide therapist ratings, the ALP provides detailed descriptors of behaviors for each observational category in each phase of learning. Observational category scores can be averaged to determine an individual’s overall phase of power mobility learning. Participants completed the NASA Task Load Index (NASA-TLX; Hart and Staveland, 1988), modified for pediatric respondents (Laurie-Rose et al., 2017). Participants rated 8 perceived workload items on a 5-point Likert scale, with higher numbers indicating greater perceived workload. Caregivers rated CYP and service provider engagement in the session using the Pediatric Rehabilitation Intervention Measure of Engagement – Observation Version (PRIME-O; King et al., 2015), a 10-item, observational measure of affective, behavioral, and cognitive engagement indicators. All items are rated on a 5-point Likert scale from 0 to 4, with higher numbers indicating greater engagement.

### 2.8 Post-training assessment

After the final training session, participants and their caregivers repeated COPM ratings of perceived performance, importance, and satisfaction to identify progress on power mobility goals. Therapists also scored each participant’s GAS goal achievement on a 5-point scale from −2 (*Much Worse Than Expected*) to +2 (*Much Better Than Expected*) based on *a priori* criteria developed during goal setting (see above).

### 2.9 Data analysis

Due to the small sample size and considerable heterogeneity within the sample, data analysis largely employed to descriptive statistics and non-parametric tests. Individual GAS scores and changes to COPM ratings of performance and satisfaction from baseline to the end of the training were calculated. Changes to scores on the COPM and GAS from first to final training session were also compared across participants using a Wilcoxon matched pairs Signed Rank Test. The proportion of participants with clinically significant changes (>2 points) on the COPM is reported. Mean participant engagement, perceived workload, and BCI training consistency scores are also reported.

## 3 Results

### 3.1 Participant characteristics

**Figure 3:**
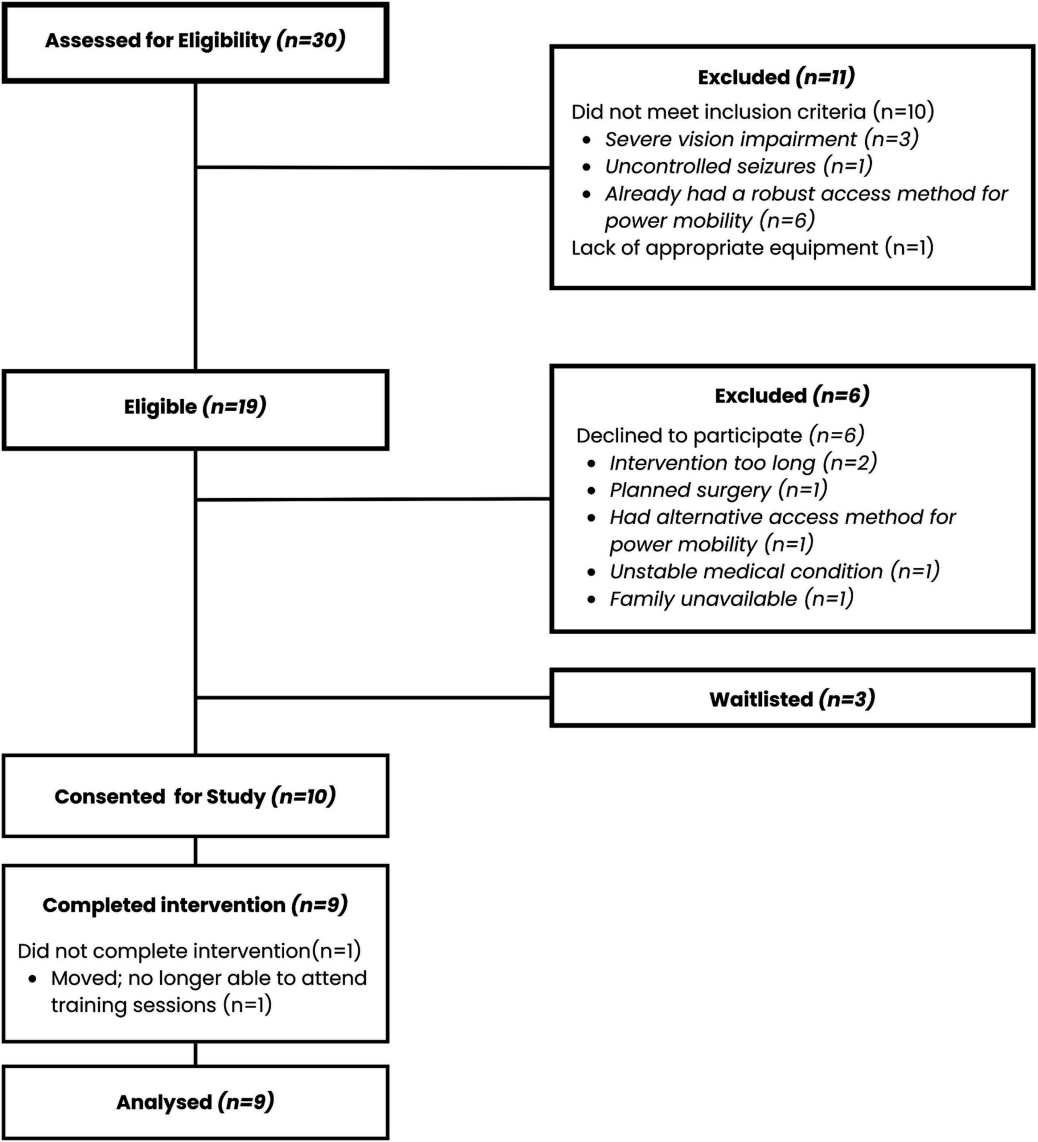
Recruitment flowchart.

Nineteen families of CYP with severe physical disabilities were invited to take part in the study. Ten families agreed to participate (recruitment rate = 52.6%). Patient numbers and reasons for declining are summarized in Figure 3. One participant consented for the study but did not complete any sessions due to a family move, leaving a sample size of nine CYP. Participants ranged in age from 7 to 17 years (mean age = 11.33 ± 3.71 years) and 3 were male (Table 1). Gender was not assumed or assigned for this study since the target population was young with complex communication disorders.

**Table 1:** Participant demographics.

Study ID	Age (yrs)	Sex	Diagnosis	CFCS^a^	GMFCS^b^	MACS^c^	Vision	Attention Span^d^	Power mobility experience
P01	10	M	Cerebral palsy	III	V	IV	Hyperopia with astigmatism, uncorrected	<45 min	None
P02	17	F	Cerebral palsy	III	V	IV	Intact	30–45 min	Yes—previously drove with two switches in a head array
P03	10	M	Cerebral palsy	V	V	V	Intact	10 min	Yes—early childhood
P04	7	F	Cerebral palsy	V	V	V	Cortical visual impairment, hyperopia corrected with glasses	1 h	Yes—early childhood
P05	17	F	Cerebral palsy	III	V	V	Intact	3–4 h	Yes—current driver with knee switch for single switch scanning
P06	11	M	Cerebral palsy	III	V	IV	Cortical visual impairment	30–60 min	None
P07	13	F	Rett syndrome	III	N/A	N/A	Intact	1 h	None
P08	10	F	Rett syndrome	IV	N/A	N/A	Mild visual impairment, corrected with glasses	30 min	None
P09	7	F	Cerebral palsy	V	IV	IV	Controlled intermittent exotropia	30–45 min	None

GMFCS, Gross Motor Function Classification System; MACS, Manual Ability Classification System.

a CFCS, Communication Function Classification System (Hidecker et al., 2011). I = Independently and effectively alternates between being a sender and receiver of information with most people in most environments; V = Seldom able to communicate effectively, even with familiar people.

b GMFCS, Gross Motor Function Classification System (Palisano et al., 1997). I = Able to walk independently in all settings; V = Transported in a manual wheelchair in all settings with limited head and trunk postures and control of leg and arm movements.

c MACS, Manual Ability Classification System (Eliasson et al., 2006). I = Handles objects easily and successfully; V = Does not handle objects and has severely limited ability to perform even simple actions.

d Reported by parent/caregivers, “How long would you estimate that your child can attend to an activity?”.

All participants had tried BCI within a clinical BCI program before starting the study, however the extent of their experience was variable (range = 0–3 years). Four participants (P01, P02, P03, P04) had previous experience with BCI-enabled power mobility in a previous pilot study (Floreani et al., 2022b). Four participants (P02, P03, P04, P05) had experience driving PMDs using other access methods. P03 and P04 had both driven switch-operated, ride-on PMDs for <1 year before 5 years of age. P02 had previously driven a PMD using two switches in a head array. P05 was a current driver when starting the study, using a knee switch for single switch scanning and was interested in using a hybrid BCI-switch system to increase the number of simultaneous PMD commands they could control. The remaining five participants (P01, P06, P07, P08, P09) had no experience driving PMDs.

Participants completed an average of 11 sessions (range = 8–12, SD = 1.41). Five participants completed all 12 power mobility training sessions. P02 completed 11 sessions due to family availability. P07 completed 8 sessions due to illness, and P08 completed 10 sessions due to family availability. Data is reported for all sessions completed for all participants.

### 3.2 Achievement of personalized power mobility goals

All participants set at least one personalized power mobility goal using the COPM. P09 set two personalized power mobility goals. Participants’ personalized power mobility goals are presented in Table 2.

**Table 2:** Participants’ personalized power mobility goals.

Study ID	Participant goals
P01	Play Star Wars & Harry Potter games
P02	Drive in the mall
P03	Move independently short distances toward people/objects/targets
P04	Play games like tag with friends in the gym
P05	Move different directions while using current access method (knee switch) to drive forward
P06	Explore and enjoy mobility independence
P07	Drive forward 50 m independently
P08	Chase friends/siblings while moving forward independently
P09	Learn driving skills without motor demands
	Move in any direction with siblings present

Personalized power mobility goals set by participants and their families using the COPM.

**Figure 4:**
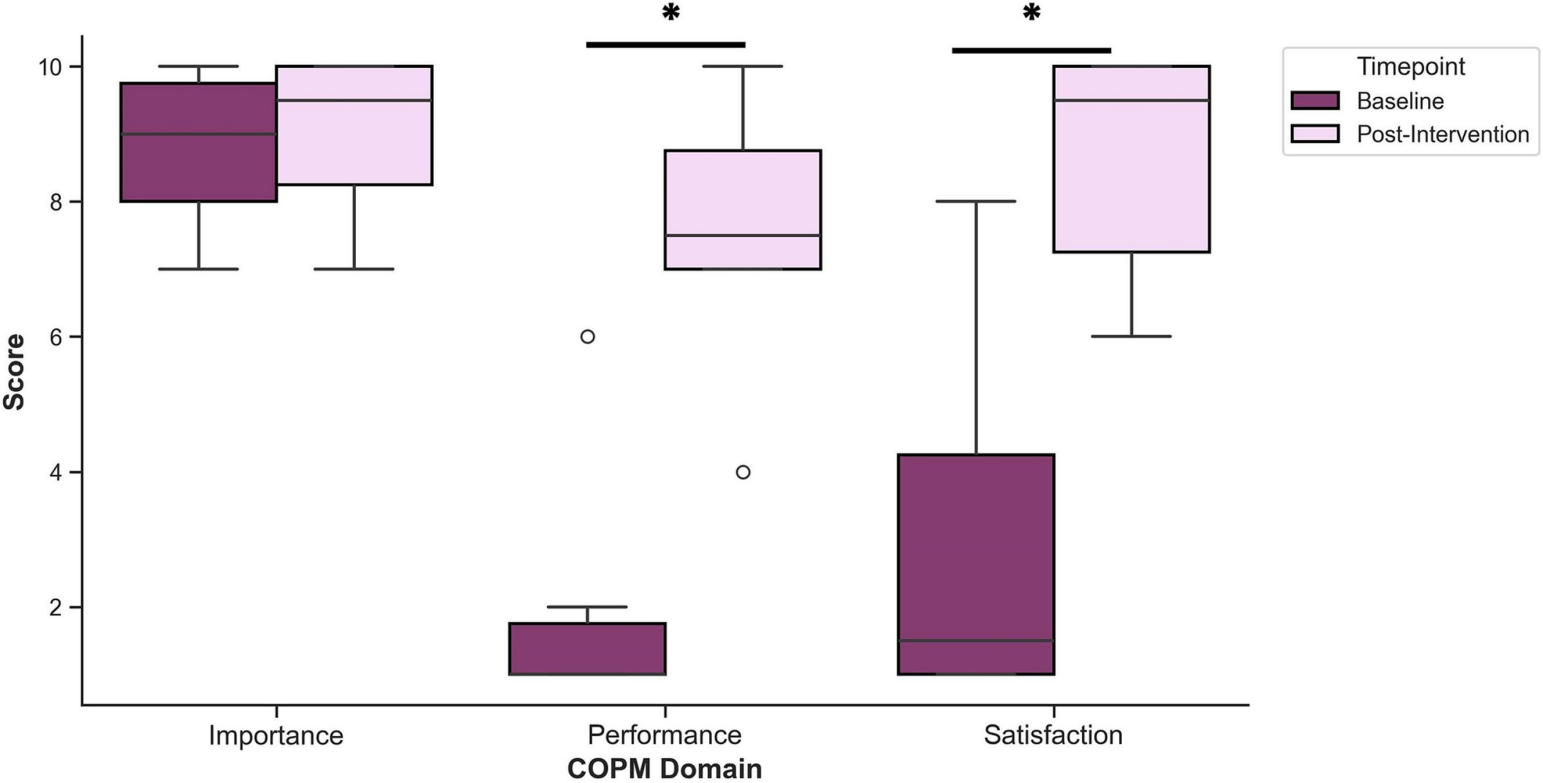
Mean COPM scores for performance, satisfaction, and importance before (baseline) and after (post-intervention) the intervention. A Wilcoxon matched-pairs signed rank test revealed significant increases to performance and satisfaction following the intervention, compared to baseline. *p < 0.05.

COPM performance and satisfaction scores for power mobility goals showed clinically significant improvements (change of ≥2 points) for all participants from baseline to post-intervention (Figure 4). Baseline performance scores ranged from 1 to 6 with a mean of 1.70 (SD = 1.57), whereas post-intervention performance scores ranged from 4 to 10 with a mean of 7.70 (SD = 1.77). Similarly, baseline satisfaction scores ranged from 1 to 8 with a mean of 2.80 (SD = 2.57), whereas post-intervention satisfaction scores ranged from 6 to 10 with a mean of 8.70 (SD = 1.57). Baseline importance scores were quite high, ranging from 7 to 10 with a mean of 8.70 (SD = 1.16). Post-intervention importance scores were similarly high, ranging from 7 to 10 with a mean of 9.10 (SD = 1.10). A Wilcoxon matched-pairs signed ranks test revealed significant differences between baseline and post-intervention COPM performance (*Z* = −2.869, adjusted *p* = 0.012) and satisfaction scores (*Z* = −2.809, adjusted *p* = 0.015). Differences between baseline and post-intervention COPM importance scores were not significant (*Z* = −1.069, adjusted *p* = 0.855).

**Figure 5:**
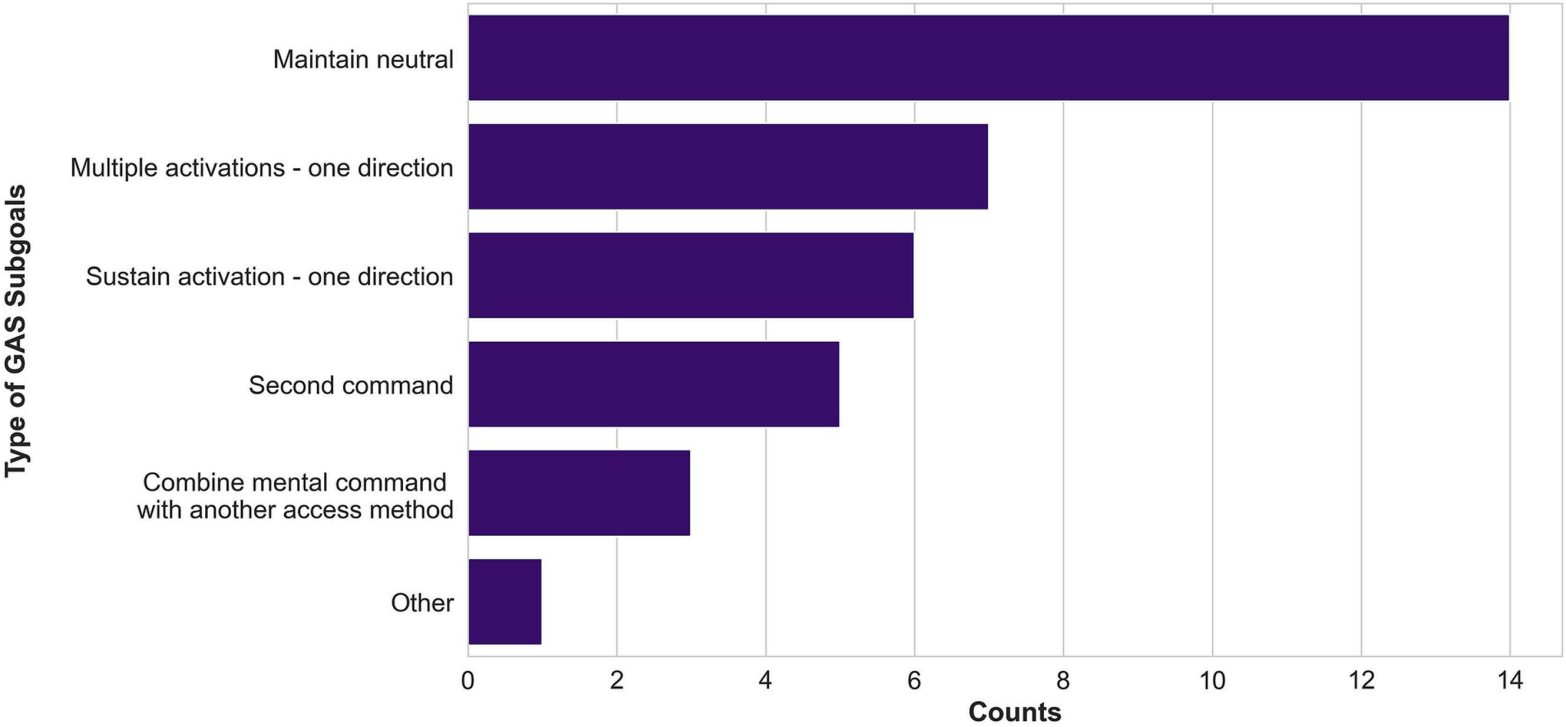
Types of GAS subgoals defined by therapists. Subgoals about maintaining the neutral command, moving in one direction with multiple activations of a mental command, and moving one direction with a sustained activation of a mental command were the most common.

Occupational therapists broke down each personalized power mobility goal into objective, subgoals using GAS. In total, 36 subgoals were identified, ranging from 2 to 4 subgoals per personalized power mobility goal (*M* = 3.6; SD = 0.7). Figure 5 displays the types of GAS goals set for study participants. Over the course of training, 88.9% of GAS goals (32/36) were met or exceeded. Baseline GAS T scores ranged from 21.0 to 37.6, with a mean of 25.30 (SD = 5.31), whereas post-intervention GAS T scores ranged from 50.0 to 77.4, with a mean of 63.42 (SD = 10.78). A Wilcoxon matched-pairs signed ranks test revealed significant differences between baseline and post-intervention GAS T scores (*Z* = 2.805, *p* = 0.005).

### 3.3 Power mobility skill acquisition

At training session 1, overall ALP scores ranged from 2 (*Curious Novice*) to 3 (*Beginner*; *M* = 2.44; SD = 0.53). Following each participant’s final training session, overall ALP scores ranged from 3 (*Beginner*) to 5 (*Sophisticated Beginner*; *M* = 3.56; SD = 0.73). Over the course of the intervention, 7 of the 9 participants’ overall ALP scores increased (77.8%), while scores for the remaining two participants remained the same (22.2%). Both participants whose scores did not change had an overall ALP score of 3 at their first and final sessions, which was similar to other participants in our sample. However, a score of 3 is the final score in Stage 1 of the ALP (*Exploration of Function*).

We performed a secondary analysis of our data comparing participants who remained in the first stage of the ALP (*overall ALP score 1–3 at final session*) to participants who progressed to the second stage (*overall ALP score 4–6 at final session*). Participants who progressed into the Stage 1 by the end of the intervention tended to be younger (*Stage 1: M* = 8.8 years, SD = 1.6 years; *Stage 2: M* = 14.5 years, SD = 3.0 years), were more likely to have visual impairments (*Stage 1:* 1/5 had intact vision; *Stage 2:* 3/4 had intact vision), had greater communication difficulties (*Stage 1:* 4/5 had CFCS IV/V; *Stage 2:* 0/4 had CFCS IV/V), and were less likely to be able to follow multistep verbal directions (*Stage 1:* 1/5; *Stage 2:* 3/4), compared to participants who remained in Stage 1 by the end of the intervention. In contrast, there were no clear differences in disease severity (*Stage 1:* 4/5 GMFCS IV/V, 4/5 MACS IV/V; *Stage 2:* 3/4 GMFCS IV/V, 3/4 MACS IV/V), or previous power mobility experience (*Stage 1:* 2/5 had previous power mobility experience; *Stage 2:* 2/4 had previous power mobility experience) between these two groups of participants.

**Figure 6:**
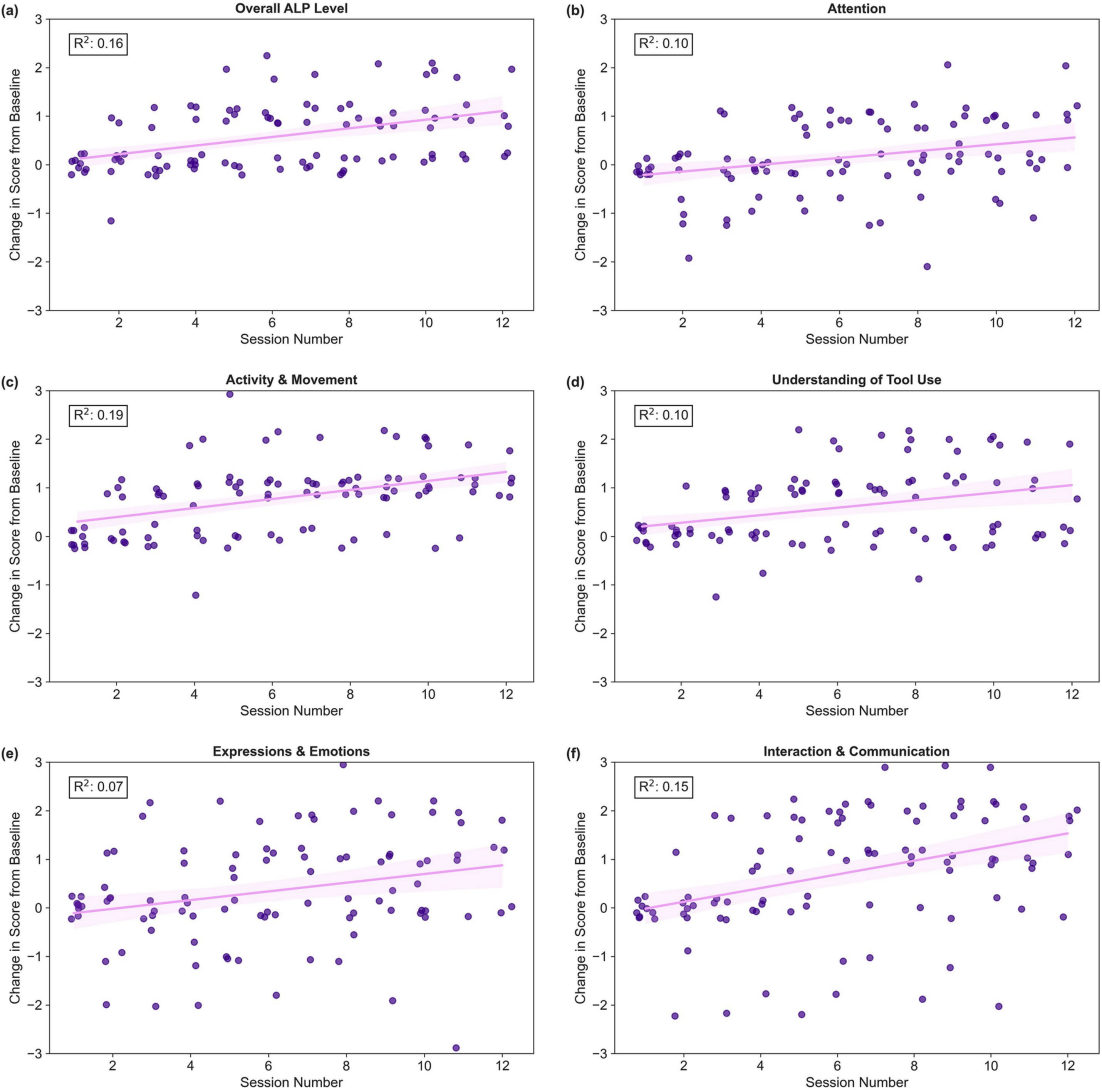
Scatter plots of changes to ALP scores from baseline by training session number with fitted regression line. Each dot represents a participant’s change in ALP score from their own baseline at a single training session. The purple line displays the least squares linear regression line with a 95% confidence interval. R-squared values are annotated in each plot. Panel **(a)** reflects overall ALP phase of learning, whereas panels **(b–f)** display ALP observational category scores, where **(b)** Attention, **(c)** Activity & Movement, **(d)** Understanding of Tool Use, **(e)** Expressions & Emotions, and **(f)** Interaction & Communication.

We conducted six simple linear regressions to explore the relationship between training session number and changes to ALP scores from baseline (overall + five observational categories). Regression revealed that changes to ALP scores from baseline had a small but statistically significant association with training session number (*R*^2^ = 0.07–0.19; adjusted *p* = <0.001–0.039). Key statistics for all six regressions are presented in Table 3. Changes to participants’ ALP scores from Session 1 are displayed in Figure 6.

**Table 3:** Summary of linear regression results for changes to alp scores from baseline.

Dependent variable	Intercept	Slope	Standard error	95% CI	*R* ^2^	*p*-value
Overall ALP	0.04	0.09	0.02	0.05–0.13	0.16	**<0.001**
Attention	−0.28	0.07	0.02	0.03–0.11	0.10	**0.001**
Activity & Movement	0.21	0.09	0.02	0.05–0.13	0.19	**<0.001**
Understanding of Tool Use	0.12	0.08	0.02	0.03–0.12	0.10	**0.001**
Expressions & Emotions	−0.20	0.09	0.03	0.03–0.15	0.07	**0.006**
Interactions & Communication	−0.16	0.14	0.03	0.07–0.20	0.15	**<0.001**

Summary table showing the results of linear regression analyses conducted on changes to ALP overall and category scores from baseline. For each regression, intercept, slope, standard error of the slope, 95% confidence interval, coefficient of determination (*R*^2^), and *p*-value are presented. A Bonferroni correction was applied to control for multiple comparisons, adjusting the significance level to 0.008. Significant *p*-values are bolded.

### 3.4 Practicality and feasibility

#### 3.4.1 BCI set-up and calibration

Across all training sessions, average time to set up the BCI headset was 7 min, 46 s (SD = 4 min, 9 s; Range = 1:00–21:41 min). We observed a trend toward shorter set-up times throughout the course of the intervention (First Session: Mean = 10:11 min, SD = 5:16 min; Final Session: Mean = 7:02 min, SD = 2:25 min). Headset setup time was slightly faster on average for the headband-style Emotiv EPOC X (Mean = 7:10 min, SD = 3:27 min), compared to the cap-style Emotiv Flex (Mean = 8:01 min, SD = 4:25 min).

Average BCI calibration time across all sessions was 16 min, 21 s (SD = 6 min, 54 s; Range – 3:17–42:00). There was no clear change in calibration times throughout the course of the intervention (First Session: Mean = 15:48 min, SD = 3:09 min; Final Session: 16:24 min, SD = 5:06 min). Longer calibration times were observed for sessions where participants calibrated more than one mental command. Factors that increased calibration time included number of commands calibrated, technical issues (e.g., software glitches, batteries dying), environmental distractions (e.g., presence of siblings, cold temperatures, noise during calibration), and poor participant mood (e.g., fatigue from previous activities or lack of sleep, illness). In 10 sessions (10.1% of total completed sessions), calibration was restarted due to excessive movement during calibration or inability to generate a consistent mental command. Participants’ mental commands and the cues used to prompt their mental commands are presented in Table 4.

**Table 4:** Participant neutral and mental commands and cues.

Study ID	Neutral command	Mental command and cues				
		Direction	Mental command	Visual cues	Verbal cues	Sessions used
P01	Imagine being Peter Parker	Forward	Imagine [being] Spiderman			1, 5–12
		Left	Imagine kicking left foot			2–4, 6–8
P02	Counting backwards from 10	Left	Imagine pushing	Red ball	“Push with your eyes”	1–11
		Reverse	Imagine jumping	Pink ball	“Jump”	3–6, 8–11
P03	Counting backwards from 10	Forward	Imagine pushing	Green ball	“Push push push”	1–5,7–12
		Right	Imagine kicking right foot	Soccer ball	“Kick kick kick”	6
P04	Five Little Ducks song	Forward	Imagine pushing	Green ball	“Go go go”	1–12
P05	Counting backwards from 10	Right	Imagine taking candy with right hand	Yellow ball	“Gebdi”	1–3, 6–10
		Left	Imagine sticking out tongue	Blue ball	“Tongue”	4–10
P06	Counting backwards from 10	Forward	Imagine squeezing a ball	Hand squeezing a ball	“Think squeeze”	1–12
		Right	Imagine kicking a ball	Picture of sibling kicking a ball	“[Sibling] kicking”	8–9, 11–12
P07	Thinking about nothing	Forward	Imagine pushing	Hands pushing away	“Go go go”	1–9
		Right	Imagine turning		“Turn turn turn”	7
		Reverse	Back back back		“Back back back”	2
P08	Sleepy brain	Forward	Imagine hitting with a unicorn wand	Pool noodle “wand”	“Hit”	1–10
P09	Sleeping brain	Forward	Imagine pushing family	Hands pushing away	“Push”	1–12
		Left	Imagine turning left		“Turn turn turn”	5–7

**Figure 7:**
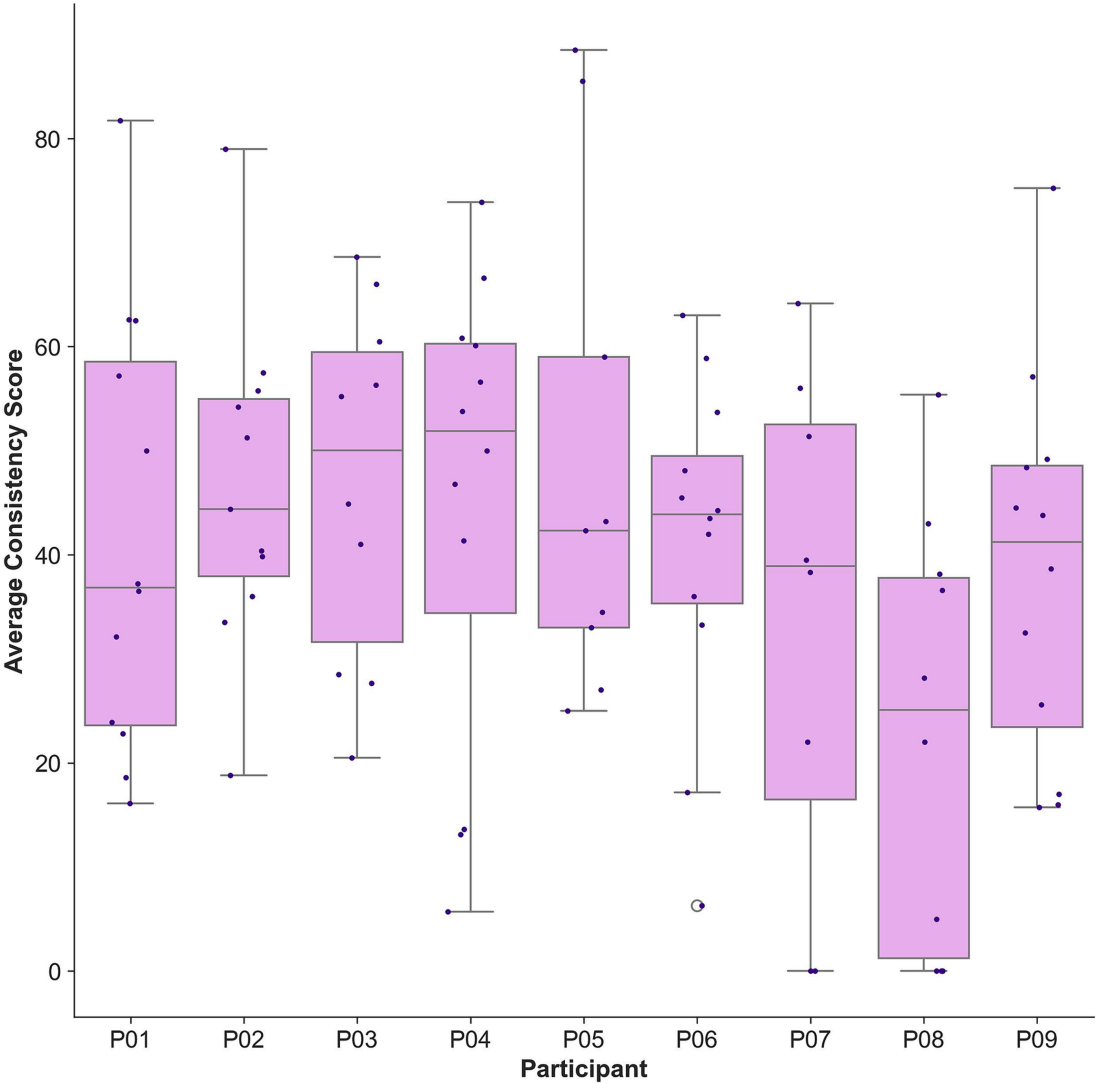
Box plot of mean calibration consistency scores by participant. Each dot represents a participant’s mean calibration consistency score at a single training session. Data for one training session was missing for participant P05 due to a technical software issue.

Participants displayed considerable variability with regards to calibration consistency scores (Figure 7). Mean calibration consistency across all sessions was 40.26% (SD = 30.32%), ranging from 24.69 to 48.54%. Across all sessions, mean minimum consistency score was 9.38% (SD = 13.28%) and mean maximum consistency score was 77.38% (SD = 22.55%). Within session variability was large, with standard deviations ranging from 1.67 to 42.34% (Figure 7).

#### 3.4.2 Perceived workload

At training session 1, overall NASA-TLX scores ranged from 1.63 (*Not Working Very Hard*) to 5.00 (*Working Very Hard*; *M* = 2.86; SD = 1.02). Following each participant’s final training session, overall NASA-TLX scores ranged from 1.63 to 3.50 (*M* = 2.56; SD = 0.61). Between participants’ first and final sessions, raw overall NASA-TLX scores decreased, indicating reduced workload, for 4/8 participants (50.0%). Raw overall NASA-TLX scores increased from first to final session for 3/8 participants (37.5%) and stayed the same for 1/8 participants (12.5%).

**Figure 8:**
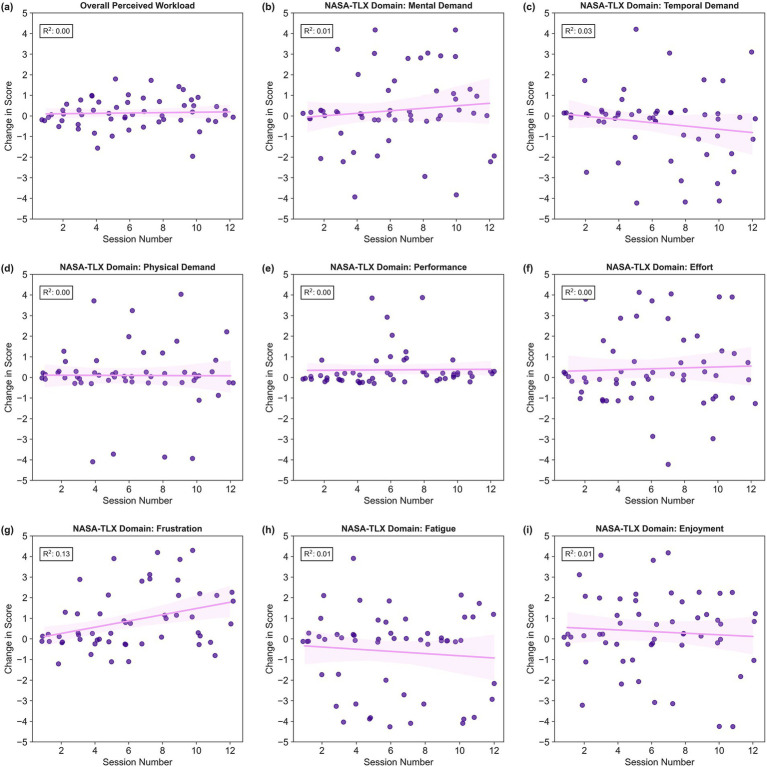
Scatter plots of changes to NASA-TLX scores from baseline by training session number with fitted regression line. Each dot represents a participant’s change in NASA-TLX score from their own baseline at a single training session. The purple line displays the least squares linear regression line with a 95% confidence interval. R-squared values are annotated in each plot. Panel **(a)** reflects overall perceived workload, whereas panels **(b–i)** display NASA-TLX domain scores, where **(b)** Mental Demand, **(c)** Temporal Demand, **(d)** Physical Demand, **(e)** Performance, **(f)** Effort, **(g)** Frustration, **(h)** Fatigue, and **(i)** Enjoyment.

We conducted nine simple linear regressions to explore the relationship between training session number and changes to NASA-TLX scores from baseline (overall + eight domain scores). Regression revealed that changes to NASA-TLX scores from baseline had no significant association with training session number (*R*^2^ = 0.00–0.13; adjusted *p* = 0.006–1.000), however there was a trend toward increased frustration over time (*R*^2^ = 0.13; adjusted *p* = 0.006). Key statistics for all nine regressions are presented in Table 5. Changes to participants’ NASA-TLX scores from Session 1 are displayed in Figure 8.

**Table 5:** Summary of linear regression results for changes to NASA-TLX scores from baseline.

Dependent variable	Intercept	Slope	Standard error	95% CI	*R* ^2^	*p*-value
Average perceived workload	0.09	0.01	0.03	−0.05 to 0.07	0.00	0.755
Mental demand	−0.12	0.06	0.07	−0.09 to 0.21	0.01	0.415
Temporal demand	0.15	−0.08	0.07	−0.22 to 0.06	0.03	0.244
Physical demand	0.11	0.00	0.06	−0.13 = 0.12	0.00	0.961
Performance	0.34	0.00	0.04	−0.07 to 0.08	0.00	0.903
Effort	0.28	0.02	0.08	−0.13 to 0.17	0.00	0.768
Frustration	−0.04	0.15	0.05	0.04–0.26	0.13	0.007
Fatigue	−0.29	−0.05	0.08	−0.22 to 0.11	0.01	0.517
Satisfaction	0.59	−0.04	0.08	−0.19 to 0.11	0.01	0.602

Summary table showing the results of linear regression analyses conducted on changes to NASA-TLX overall and category scores from baseline. For each regression, intercept, slope, standard error of the slope, 95% confidence interval, coefficient of determination (*R*^2^), and *p*-value are presented. A Bonferroni correction was applied to control for multiple comparisons, adjusting the significance level to 0.006. Significant *p*-values are bolded.

#### 3.4.3 Participant engagement

Participants and service providers were highly engaged in all training sessions (Mean PRIME-O Score = 3.77; SD = 0.44). Mean scores for affective (3.75; SD = 0.48), behavioral (3.94; SD = 0.21), and cognitive (3.76; SD = 0.42) components of engagement were highly consistent. Mean PRIME-O domain scores from each session can be viewed in Supplementary material 2.

## 4 Discussion

This study aimed to assess whether BCI can be used by CYP with severe physical disabilities to achieve individualized, functional power mobility goals. Our results support this hypothesis and highlight that BCI can help CYP with disabilities achieve personalized power mobility goals and acquire power mobility skills. This work also supports the feasibility of BCI-enabled power mobility training for CYP with significant motor impairments, demonstrating favorable results in terms of perceived workload, session engagement, headset and calibration tolerability, and successful BCI use. To the best of our knowledge, this study is the first-of-its-kind exploring clinical implementation of a BCI for CYP with physical disabilities exploring power mobility, building on previous findings outlined in Floreani et al. (2022b). Overall, our results highlight that BCI may be a viable power mobility access method for CYP with physical disabilities, but that BCI performance optimization and BCI skill acquisition may be needed to help translate this technology into clinical use.

### 4.1 Personalized power mobility goal achievement

Personalized power mobility goal performance and satisfaction improved significantly over the course of the intervention. This suggests that the intervention positively impacted participants’ overall experience and perception of their own abilities. Clinically significant improvements (a change of ≥2 points) were observed for all participants, even those with relatively high baseline performance and satisfaction ratings, which likely reflects a substantial perceived benefit of adding BCI to existing access methods for participants who had limited PMD control with another access method prior to the intervention. Interestingly, we observed notable goal achievement despite limited improvement of BCI skills, as suggested by calibration consistency scores. This is consistent with findings from Kelly et al. (2023), who found that, after using BCI at home for a 12-week period, families reported dramatic improvements in personalized goal performance and satisfaction, despite limited changes to BCI calibration consistency.

Additionally, we observed significant improvements to therapist-rated GAS goals. Therapist-rated GAS goals were scored based on observable features and were scaled *a priori*, which suggests that goal achievement reflected some actual changes in driving abilities, not just perceived improvements. Again, given that these changes occurred in the absence of clear BCI skill development suggests that modifying the intervention or BCI system to optimize BCI learning could harness greater functional impacts for participants.

### 4.2 Power mobility skill acquisition

We observed a trend toward small improvements to ALP scores over time, which may indicate that the intervention supported gradual development of power mobility skills. These results are consistent with recent findings from Floreani et al. (2022b), who reported that children with quadriplegic cerebral palsy displayed variable changes to power mobility skills across two BCI-enabled power mobility training sessions. However, independent of BCI, the development of power mobility skills is highly variable, and some CYP may never progress from using power mobility in limited environments with constant assistance and close supervision (Field and Livingstone, 2018). The trends observed may reflect a challenge with power mobility skill acquisition inherent to our sample, rather than a consequence of the access method selected for this study. Although session-to-session changes in ALP scores were relatively mild, any progression in ALP scores is an encouraging finding given that most of our participants were previously unable to reliably control a PMD using alternative access methods. Even exploratory independent mobility can have positive developmental impacts for CYP with significant motor impairments (Rosen et al., 2018).

It is also difficult to comment on the speed of learning compared to other power mobility access methods. For example, children with complex conditions may acquire power mobility skills more slowly than children with milder impairments and, in some cases, may never progress beyond exploratory PMD control (Field and Livingstone, 2018). Previous case series have reported similar changes to ALP scores for children with severe physical disabilities, improving 1–3 phases over several months following power mobility interventions using other access methods (Kenyon et al., 2023; Kenyon et al., 2021; Livingstone and Field, 2023). Trends from our data may suggest that age, communication abilities, visual function, and direction following may be important factors for BCI-enabled power mobility skill acquisition.

The variability in previous power mobility experience within our sample adds complexity to our results. Experience with power mobility can have broad developmental impacts for children with mobility limitations, including improvements to cognition and receptive language (e.g., Jones et al., 2012). There was a slight tendency for participants with previous power mobility experience to have higher post-intervention ALP scores, particularly in the domains of Activity & Movement and Understanding of Tool Use, despite similar baseline ALP scores. Time spent in a power mobility device is correlated with greater power mobility competence, regardless of cognitive abilities or severity of motor impairment (Bottos et al., 2001). Power mobility training can also facilitate understanding of cause-effect relationships for individuals with significant motor impairments (Nilsson and Nyberg, 1999; Nilsson et al., 2011). Participants with previous power mobility experience in this study may have developed a better baseline understanding of cause-effect relationships through their previous power mobility experiences, allowing them to quickly progress to intentional control of the power chair, despite limited familiarity with BCI-enabled power mobility.

Further study is required to compare power mobility skill acquisition using BCI to skill alternative access methods and to determine what degree of power mobility proficiency will be achievable for CYP with significant physical disabilities using BCI.

### 4.3 Practicality and feasibility of BCI-enabled power mobility

#### 4.3.1 BCI set-up and calibration consistency

There was a trend toward shorter BCI set-up times throughout the course of the intervention. This was likely related to increased efficiency of by the investigators as they became more familiar with the set up and better able to navigate barriers (e.g., hairstyles, wheelchair headrests, head shape, involuntary movements, etc.). This change may also reflect increased participant comfort and familiarity with the set-up process. In contrast, there was no clear change to calibration times, over the course of the intervention. This is difficult to interpret. Although all participants began the intervention training a single BCI command, most began training two BCI commands at some point throughout the intervention. We only measured total calibration time, so we were not able to measure changes to calibration time for each command over time.

BCI calibration consistency scores were highly variable and did not show a tendency to improve over time. These results are consistent with previous longitudinal research conducted on BCI use among CYP with physical disabilities. Kelly et al. (2023) reported BCI calibration consistency among children with quadriplegic cerebral palsy who used BCI at home over a 12 weeks. In that study, calibration consistency scores varied widely both within and across participants, with no significant improvements across sessions (Kelly et al., 2023). This variability likely reflects the complexity of BCI use among CYP with neuromotor impairments. Multiple factors introduce variability between individuals and between sessions, including brain anatomy and functional organization, stage of neurodevelopment, attention, engagement, and differences in mental strategies used (Huggins et al., 2022; Saha and Baumert, 2020). However, variability in calibration consistency may also indicate that the proprietary classification algorithms in the Emotiv Cortex API were not well suited to application in pediatric BCI (Orlandi et al., 2021).

#### 4.3.2 Perceived workload

In this study, perceived workload was moderate and remained relatively stable over time. This suggests that perceived workload did not change, despite gradually improving power mobility skills. Participants’ training plans were thoughtfully designed to ensure that activities were challenging and engaging throughout the intervention. Therefore, stability of participants’ perceived workload may reflect successful scaling of session activities by the study clinicians. However, it may be that the stability of perceived workload was related to a lack of progression in BCI skills. BCI control can be developed through practice and focused experience (McFarland and Wolpaw, 2018). However, participants’ calibration consistency scores displayed considerable variability across sessions with no tendency toward improvement over time, although given the lack of clarity surrounding how calibration consistency scores are calculated, it is difficult to comment on participants’ BCI skills. As discussed above, optimizing classification algorithms for pediatric users may further enable improved BCI skills development. It is also possible that a greater focus on training BCI skills before or during the intervention may have reduced participants’ perceived workload. Further research on long-term BCI skill acquisition and algorithm optimization for pediatric users with severe physical disabilities is needed to fully interpret these findings.

The trend of stable, moderate perceived workload was consistent across all NASA-TLX subscales, except for frustration, which tended to increase over time. In the absence of other changes to workload, there are several possible explanations for increased frustration over time. Although the NASA-TLX does not effectively capture why participants were feeling frustrated, anecdotally, many caregivers reported that participants were frustrated by technical issues experienced throughout the intervention. Previous research suggests that users may experience greater levels of frustration with technology when technical issues recur and when users have low perceived control over the issue (Hertzum and Hornbæk, 2023). Even though the frequency of technical issues was relatively stable over time, users may have experienced greater frustration as technical issues recurred throughout the intervention. Though optimizing the technology to mitigate disruptive issues would likely alleviate frustration, lowering user expectations for the technology used in sessions may also reduce frustration (Ferreri and Mayhorn, 2023). It is possible that the intervention was too long for participants. Breaking the intervention up several shorter bursts may help mitigate frustration for participants and keep the intervention engaging and motivating.

#### 4.3.3 Engagement

Families and therapists were highly engaged in BCI-enabled power mobility training. Unfortunately, we observed a clear ceiling effect on the PRIME-O, which limited our ability to draw more nuanced conclusions regarding engagement. We plan to supplement this data with qualitative analysis of post-intervention interviews, which were completed by families but are not reported in this article, and possibly with a post-study family focus group. However, these results reflect a strong interest by families to try new access methods for power mobility. These results are consistent with qualitative reports from families regarding their participation in a clinical BCI program, who indicated that taking part in the program was exciting and engaging, a sentiment that was bolstered when children experienced new forms of independence (Jadavji et al., 2021).

### 4.4 Limitations

There were several notable limitations to this study. First, the small sample size limited the statistical analyses we were able to conduct. Although we recruited from two separate sites, it is possible that recruiting through the clinical BCI programs introduced some bias into our sample. Families who chose to participate in the BCI program at either site may have been more likely to be successful than CYP with similar impairments who did not participate in this optional program. The study sample was highly heterogenous in terms of clinical characteristics, personalized power mobility goals, activities completed throughout the intervention, and previous experience with BCI and power mobility experience. For example, variability in the difficulty of personalized power mobility goals may have affected feelings of perceived workload and, ultimately, goal achievement and power mobility skill acquisition. Personalized goal setting was facilitated by clinicians with extensive experience using BCI, who helped patients and families to select goals that were appropriately challenging for their baseline skills. We did our best to control for individual variability by using each participant as their own control when comparing perceived workload over the course of the intervention. However, this variability reflects the diverse characteristics of CYP with severe motor impairments, perhaps supporting generalizability to larger populations. Despite this variability, the observed improvements to satisfaction and performance of personalized power mobility suggest broad potential for BCI-enabled power mobility for CYP with physical disabilities.

Another limitation was the selected BCI headset and associated software. We chose to use Emotiv headsets based on their suitability for pediatric users (i.e., size of headset, comfort, time to don/doff, etc.; Jadavji et al., 2022). However, Emotiv’s classification algorithms are proprietary, and the organization offers limited transparency on methodological details. Although the Emotiv Cortex API software provides a “consistency score” for each calibration trial, it is unclear how this metric is calculated. Therefore, it is difficult to draw any clear conclusions about BCI skill development in this study.

There were also limitations regarding the outcome measures selected for this study. We observed considerable ceiling effects on the PRIME-O. While participants were highly engaged in the intervention, a more robust measure may have detected greater nuance. It is also challenging to determine the reliability of responses to the NASA-TLX. Although NASA-TLX is commonly used to measure mental workload in BCI studies, many of the CYP in this study experienced communication difficulties, even when using alternative access methods. Both caregivers and clinicians in this study noted that participant responses to the NASA-TLX did not always align with what was observed during training sessions. Finally, we used Goal Attainment Scaling to measure progress toward personalized power mobility goals. Although Goal Attainment Scaling can detect meaningful changes in pediatric rehabilitation, further standardization of GAS administration and scoring would have added further rigor to this study’s methodology (Angeli et al., 2019).

### 4.5 Conclusion

This study demonstrated that simple, commercially available BCI systems could be harnessed to help CYP with severe physical disabilities achieve individualized, functional power mobility goals. Participants displayed a trend toward improved power mobility skills over time and were highly engaged throughout. BCI calibration consistency and perceived workload did not change over time. The development of specific BCI skill acquisition strategies and the optimization of BCI classification algorithms for pediatric users, may further augment the impact of training for BCI-enabled PMD control. Overall, these results suggest that BCI may be a promising power mobility access method for CYP with significant motor impairments.

## Data Availability

The raw data supporting the conclusions of this article will be made available by the authors without undue reservation.
