# Supplementary material for: BCI move: exploring pediatric BCI-controlled power mobility
Source: Front Hum Neurosci. 2025 Apr 9;19:1456692. doi: 10.3389/fnhum.2025.1456692 (PMC12015940; doi:10.3389/fnhum.2025.1456692)
Supplement: Supplementary file 1 [file Table_1.DOCX]

Supplementary Material

# Supplemental Tables

| **SUPPLEMENTAL TABLE 1: Sample Goal Attainment Scaling** | | | | | |
| --- | --- | --- | --- | --- | --- |
| **Subgoal** | **Score** | **Criteria** | **Weight** | **Baseline Score** | **Achieved Score** |
| Move forward 5m using repeated mental commands in 4 sessions | -2 | Move forward 1m using repeated mental commands in 4 sessions | 1 | -1 | 1 |
|  | -1 | Move forward 3m using repeated mental commands in 4 sessions |  |  |  |
|  | 0 | Move forward 5m using repeated mental commands in 4 sessions |  |  |  |
|  | 1 | Move forward 7m using repeated mental commands in 4 sessions |  |  |  |
|  | 2 | Move forward 9m using repeated mental commands in 4 sessions |  |  |  |
| Move forward 1-3m using a single mental command in 4 sessions | -2 | Move forward 1-3m using a single mental command in 0 sessions | 1 | -2 | 0 |
|  | -1 | Move forward 1-3m using a single mental command in 2 sessions |  |  |  |
|  | 0 | Move forward 1-3m using a single mental command in 4 sessions |  |  |  |
|  | 1 | Move forward 1-3m using a single mental command in 6 sessions |  |  |  |
|  | 2 | Move forward 1-3m using a single mental command in 8 sessions |  |  |  |
| Maintain a stationary position by keeping mental command strength below threshold for 10s in 5-6 sessions | -2 | Keep mental command below threshold for 10s in 1-2 sessions | 1 | -1 | -1 |
|  | -1 | Keep mental command below threshold for 10s in 3-4 sessions |  |  |  |
|  | 0 | Keep mental command below threshold for 10s in 5-6 sessions |  |  |  |
|  | 1 | Keep mental command below threshold for 10s in 7-8 sessions |  |  |  |
|  | 2 | Keep mental command below threshold for 10s in 9-10 sessions |  |  |  |
| Use a second mental command to turn in 4 sessions | -2 | Use a second mental command to turn in 0 sessions | 1 | -2 | 0 |
|  | -1 | Use a second mental command to turn in 2 sessions |  |  |  |
|  | 0 | Use a second mental command to turn in 4 sessions |  |  |  |
|  | 1 | Use a second mental command to turn in 6 sessions |  |  |  |
|  | 2 | Use a second mental command to turn in 8 sessions |  |  |  |
| *Notes:* Sample GAS data for a single study participant. Three to four subgoals were defined for each participant based on their personalized power mobility goal.  **Subgoal:** objective, observable outcomes defined by study therapists based on participants’ personalized power mobility goals.  **Score:** score value for therapist-defined criteria. GAS raw scores range from -2 to +2, where -2 indicates no change in performance from baseline, 0 is the expected outcome, and +2 is much better than the expected outcome.  **Criteria:** based on the expected outcome for the participant, defines outcomes that would be associated with each score for a subgoal.  **Weight:** product of importance and difficulty of the subgoal. Given the dearth of available evidence regarding BCI skill acquisition in this population, all subgoals were weighted equally at 1.  **Baseline Score:** rates the participant’s ability in the subgoal area before the intervention. Baseline performance was set at -2 for all subgoals where participants had no previous experience and -1 for subgoals where participants had some previous experience.  **Achieved Score:** rates the participant’s performance at the end of the intervention based on therapist-defined criteria. Achieved scores range from -2 to +2, where -2 is no change in performance from baseline, 0 is the expected outcome, and +2 is much better than the expected outcome. | | | | | |

| SUPPLEMENTAL TABLE 2: CAREGIVER-RATED TRAINING PROGRAM ENGAGEMENT | | | | |
| --- | --- | --- | --- | --- |
| Session Number | **Mean PRIME-O Engagement** | | | |
|  | **Client^a^ (SD)** | **Service Provider^b^ (SD)** | **Session^c^ (SD)** | **Overall^d^ (SD)** |
| *1* | 3.42 (0.68) | 3.93 (0.19) | 3.86 (0.38) | 3.71 (0.34) |
| *2* | 3.04 (1.40) | 3.75 (0.56) | 3.80 (0.45) | 3.19 (1.28) |
| *3* | 3.50 (0.73) | 4.00 (0.00) | 3.90 (0.22) | 3.78 (0.29) |
| *4* | 3.57 (0.72) | 4.00 (0.00) | 3.93 (0.19) | 3.85 (0.22) |
| *5* | 3.33 (1.07) | 4.00 (0.00) | 3.83 (0.41) | 3.70 (0.51) |
| *6* | 3.26 (0.84) | 4.00 (0.00) | 3.92 (0.20) | 3.70 (0.36) |
| *7* | 3.90 (0.22) | 4.00 (0.00) | 4.00 (0.00) | 3.96 (0.09) |
| *8* | 3.40 (0.95) | 3.90 (0.22) | 3.80 (0.45) | 3.68 (0.55) |
| *9* | 3.46 (0.87) | 4.00 (0.00) | 3.92 (0.20) | 3.75 (0.37) |
| *10* | 3.88 (0.21) | 4.00 (0.00) | 4.00 (0.00) | 3.95 (0.08) |
| *11* | 3.81 (0.38) | 4.00 (0.00) | 4.00 (0.00) | 3.93 (0.15) |
| *12* | 3.81 (0.24) | 4.00 (0.00) | 4.00 (0.00) | 3.92 (0.10) |
| *Note:*  ^a^ Mean client engagement calculated as the average of Q1-4 from the PRIME-O.  ^b^ Mean service provider engagement calculated as the average of Q5-8 from the PRIME-O.  ^c^ Mean session engagement calculated as the average of Q9-10 from the PRIME-O.  ^d^ Mean overall engagement calculated as the average of Q1-10 from the PRIME-O. | | | | |

# Other Supplements

## Calculation of GAS T Scores:

All of a participant’s subgoals were incorporated into the calculation of a single GAS T Score, which describes overall goal attainment. GAS T Scores are a standardized score with a mean of 50 and a standard deviation of 10. All GAS T scores were calculated using spreadsheet calculators made available from Turner-Stokes. The general equation for calculating GAS T scores is included below:

$$GAS T Score =50+ \frac{10* \sum(w_{i}x_{i})}{{[\left( 1-\rho\right)\sum{w_{i}}^{2}+ \rho{(\sum w_{i})}^{2}]}^{\frac{1}{2}}}$$

Where:

w_i_ = the weight assigned to the *i*th goal. For equally weighted subgoals, w_i_ = 1.

x_i_ = the score achieved following the intervention (ranges from -2 to +2). x_i_ can also be replaced with baseline scores to calculate a standardized score of baseline performance (baseline GAS T score).

ρ = the expected correlation of the goal scales. The GAS manual suggests using a value of 0.3 for ρ.

An example GAS T Score calculation for the sample data provided in Supplemental Table 1 is provided below:

$$GAS T Score =50+ \frac{10*[\left( 1*1 \right)+\left( 1*0 \right)+\left( 1*-1 \right)+\left( 1*0 \right)]}{{[\left( 0.7 \right)(4)+(0.3){(4)}^{2}]}^{\frac{1}{2}}}$$

$$GAS T Score =50+ \frac{0}{2.82}$$

$$GAS T Score =50$$
